# Educational efficacy of high-fidelity simulation in neonatal resuscitation training: a systematic review and meta-analysis

**DOI:** 10.1186/s12909-019-1763-z

**Published:** 2019-08-29

**Authors:** Jichong Huang, Ying Tang, Jun Tang, Jing Shi, Hua Wang, Tao Xiong, Bin Xia, Li Zhang, Yi Qu, Dezhi Mu

**Affiliations:** 1 Department of Pediatrics, West China Second University Hospital, Sichuan University, Chengdu, 610041 China; 2 Key Laboratory of Obstetric & Gynecologic and Pediatric Diseases and Birth Defects of Ministry of Education, Sichuan University, Chengdu, 610041 China; 3 Department of Ultrasound, West China Second University Hospital, Sichuan University, Chengdu, 610041 China

**Keywords:** Neonatal resuscitation, High-fidelity simulation, Training, Systematic review, Meta-analysis

## Abstract

**Background:**

The training of neonatal resuscitation is an important part in the clinical teaching of neonatology. This study aimed to identify the educational efficacy of high-fidelity simulation compared with no simulation or low-fidelity simulation in neonatal resuscitation training.

**Methods:**

The PubMed, EMBASE, Cochrane Library, ClinicalTrials.gov, Chinese databases (CBM, CNKI, WanFang, and Weipu), ScopeMed and Google Scholar were searched. The last search was updated on April 13, 2019. Studies that reported the role of high-fidelity simulation in neonatal resuscitation training were eligible for inclusion. For the quality evaluation, we used the Cochrane Risk of Bias tool for RCTs and Risk Of Bias In Non-randomized Studies of Interventions (ROBINS-I) tool for non-RCTs. A standardized mean difference (SMD) with a 95% confidence interval (CI) was applied for the estimation of the pooled effects of RCTs.

**Results:**

Fifteen studies (10 RCTs and 5 single arm pre-post studies) were ultimately included. Performance bias existed in all RCTs because participant blinding to the simulator is impossible. The assessment of the risk of bias of single arm pre-post studies showed only one study was of high quality with a low risk of bias whereas four were of low quality with a serious risk of bias. The pooled results of single arm pre-post studies by meta-analysis showed a large benefit with high-fidelity simulation in skill performance (SMD 1.34; 95% CI 0.50–2.18). The meta-analysis of RCTs showed a large benefit in skill performance (SMD 1.63; 95% CI 0.49–2.77) and a moderate benefit in neonatal resuscitation knowledge (SMD 0.69; 95% CI 0.42–0.96) with high-fidelity simulation when compared with traditional training. Additionally, a moderate benefit in skill performance (SMD 0.64; 95% CI 0.06–1.21) and a small benefit was shown in knowledge (SMD 0.39; 95% CI 0.08–0.71) with high-fidelity simulation when compared with low-fidelity simulation.

**Conclusions:**

Improvements of efficacy were shown both in resuscitation knowledge and skill performance immediately after training. However, in current studies, the long-time retention of benefits is controversial, and these benefits may not transfer to the real-life situations.

**Electronic supplementary material:**

The online version of this article (10.1186/s12909-019-1763-z) contains supplementary material, which is available to authorized users.

## Background

Prompt and effective neonatal resuscitation is an important measure for reducing mortality due to neonatal asphyxia [1]. Therefore, the training of neonatal resuscitation has been thought to be an important part in the clinical teaching of neonatology [2]. The traditional neonatal resuscitation teaching method combines an explanation of the theory with a multimedia demonstration. However, this teaching method may result in insufficient procedural proficiency and resuscitation expertise for many trainees in the real-life rescue process [3].

Simulation-based education (SBE) has been introduced into advanced life support courses [4, 5]. The traditional or low-fidelity simulation was first applied, but it was controlled by the instructor and limited to the physiological feedback. High-fidelity simulation is the computer-driven manikin that utilizes physiological and pharmacological modeling algorithms to mimic real-life situations [6], which has the characteristics of assessing physical findings, making clinical decisions, and increasing realism of interactions in a team-based resuscitation environment [7, 8]. High-fidelity manikins not only approximate preterm and full-term neonates in size and weight, but they also possess a realistic airway, skin color, pulse and other vital signs, and umbilicus with a life-like pulse that can respond to hypoxic-ischemic events and interventions controlled by integrated computer programs. These advantages provide important cues for students to accurately assess the neonate and allow practice of certain procedures, such as tracheal intubation and insertion of umbilical venous catheters in manikins [6]. It’s known that simulation is only a technique. Trainees benefit not just only from simulation but more importantly from the specific training contents such as experienced facilitators, case scenarios, and the debriefing [5]. Therefore, it is easier to implement case scenarios and provide debriefing on resuscitation training with high-fidelity simulation compared with traditional simulation. The use of high-fidelity simulation for pediatric advanced life support (PALS) training was proven beneficial for improved skill performance at course conclusion in a recent meta-analysis [9]. Nevertheless, the educational efficacy of high-fidelity simulation in neonatal resuscitation training remains controversial. Some studies found improved knowledge [10], skill performance [11], or teamwork performance [12] after high-fidelity simulation training, whereas other studies showed conflicting results [13, 14]. Thus, the objective of this systematic review and meta-analysis was to assess whether the method of high-fidelity simulation is effective in neonatal resuscitation training.

## Methods

This study was conducted according to the PRISMA (Preferred Reporting Items for Systematic Reviews and Meta-Analyses) Guidelines [15].

### Study identification and selection

The PubMed, EMBASE, Cochrane Library, ClinicalTrials.gov, Chinese databases (CBM, CNKI, WanFang, and Weipu), ScopeMed and Google Scholar were searched. The search keywords and subject terms were (“simulation” OR “manikin” OR “mannequin”) AND (“neonatal resuscitation” OR “infant resuscitation”) AND (“training” OR “teaching” OR “education”). Search terms were shown in the Additional file 1. The search was limited to English or Chinese language reports and was finally updated on April 13, 2019. The titles and abstracts of the reports were screened by three authors (JS, HW and TX) independently to determine their eligibility according to the following inclusion criteria: (a) studies that investigated the role of high-fidelity simulation in neonatal resuscitation training; (b) the training was followed the Neonatal Resuscitation Program (NRP) standard; (c) clinical trial studies; (d) outcomes assessment focusing on individual or team resuscitation performance (e.g., knowledge, skill and confidence). The following exclusion criteria were also applied after reading the full texts: (a) reviews or non-trials; (b) studies written in a non-English or non-Chinese language; (c) comparisons that did not include high-fidelity simulation and other training strategies, and (d) studies without control groups and self controls. The reference list of the included studies was also screened to ensure a comprehensive search. Any disagreements were reconciled by another author (JT) who independently reviewed the studies, and then discussed disagreements with the initial reviewers until a consensus was reached.

### Data extraction

The extracted data included the first author, publication year, country, study design, included population, sample size, comparison, outcome measures, and results. Furthermore, the details of the interventions of the included studies were extracted, including the manikins, training content, instructors, scenarios, debriefing, and learning time and duration. Two authors (JS and BX) independently collected data from each study and compared the results. Any disagreement was resolved by discussions with a third author (YT).

### Quality evaluation

The Cochrane Collaboration’s Risk of Bias tool [16] was used to assess the methodological quality of each included randomized controlled trial (RCT) based on seven domains (random sequence generation, allocation concealment, blinding of participants, blinding of outcome assessment, incomplete outcome data, selective outcome reporting, and other bias). The Risk Of Bias In Non-randomized Studies of Interventions (ROBINS-I) tool [17] was used to assess the methodological quality of non-RCTs based on seven domains (confounders, selection of participants into the study, classification of interventions, deviations from the intended intervention, missing data, measurement of outcomes, and selection of the reported result). Two authors (LZ and YQ) assessed the quality of studies independently, and disagreements between them were resolved through discussion with a third author (JH).

### Statistical analysis

RCTs reporting the same level of outcome were included in the quantitative synthesis, and a standardized mean difference (SMD) with a 95% confidence interval (CI) were used to facilitate direct comparison of the results. A fixed effect model was used when heterogeneity across studies was not detected. Otherwise, a random effect model was used. Data were considered statistically heterogeneous if *P* < 0.1 and I^2^ > 50%. A *P*-value of 0.05 indicated statistically significant differences. The clinical significance of results was classified according to Cohen’s effect size (or SMD), where SMD > 0.8 = large, SMD 0.5–0.8 = moderate, SMD 0.2–0.5 = small, and SMD < 0.2 = negligible [18]. Forest plots were used to show the SMD and 95% CIs of each individual study and the pooled effect. All statistical tests were performed using Review Manager 5.3 software.

## Results

### Study characteristics

**Fig. 1:**
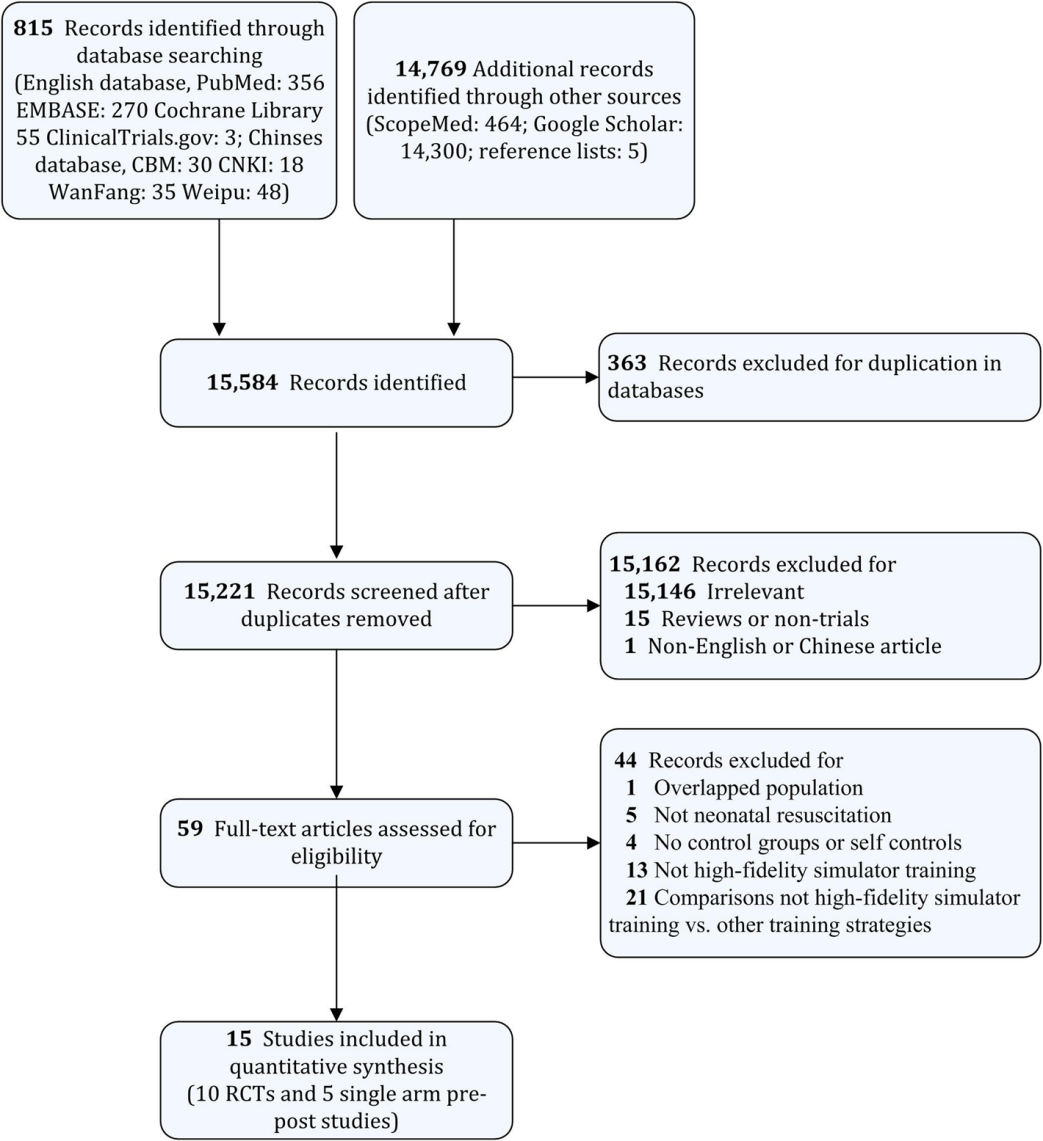
Flow diagram of the study selection process

Overall, 15,584 studies were initially identified, and 15 studies were ultimately selected. A flow diagram detailing the selection process is shown in Fig. 1. Characteristics of the included studies are summarized in Table 1. These studies were published between 2009 and 2018; five of them were conducted in Canada, five in the United States, two in China, one in India, one in Sweden, and one in France. The population consisted of residents, medical students, undergraduate students, neonatal trainees, physicians, and nurses. The sample sizes varied from 13 to 180 participants. Neonatal resuscitation knowledge, skill performance, teamwork performance, confidence survey, and satisfaction survey were outcomes after training.

**Table 1:** Characteristics of the included studies

Study	Design	Population/Sample Size	Comparison	Outcome measures	Results
Hossino et al., 2018, USA [19]	Single arm pre-post study	Residents/*n* = 26	Pre-intervention test vs. Post intervention test	Confidence survey	Improved confidence in all evaluated aspects of the survey after high-fidelity intervention, *p* < 0.01
Malmstrom et al., 2017, Sweden [20]	Single arm pre-post study	physicians, nurses and midwives/*n* = 92	Pre-intervention test vs. Post intervention test	Participants’ self-assessed questionnaire: communication, leadership, confidence and technical skills	Improved participants’ self-assessed ability to communication, leadership, confidence and technical skills, *p* < 0.001
Surcouf et al., 2013, USA [11]	Single arm pre-post study	Residents/*n* = 32	Pre-intervention test vs. Post intervention test	Knowledge, skill and teamwork performance; Confidence survey	Improved performance and confidence after high-fidelity intervention, *p* < 0.05
Finan et al., 2012, Canada [21]	Single arm pre-post study	First-year pediatric residents/*n* = 13	Pre-intervention test vs. Post intervention test	Skill performance assessed by Neonatal intubation checklist and Global rating scale	Improved skill performance scores after high-fidelity intervention in simulations test (*p* < 0.05) rather than real-life test
Sawyer et al., 2011, USA [22]	Single arm pre-post study	Pediatric and Family Medicine residents/*n* = 30 (15 teams)	Pre-intervention test vs. Post intervention test	NRP performance scores and times	Improved overall NRP performance scores and positive-pressure ventilation after high-fidelity intervention, *p* < 0.05
Wang et al., 2017, China [23]	RCT	Medical students/*n* = 180	High-fidelity simulator group (*n* = 90) vs. traditional training group (*n* = 90)	Knowledge test; Skill performance test; Satisfaction survey	Improved knowledge scores and skill performance in high-fidelity group, *p* < 0.001; Improved satisfactory in learning theoretical knowledge, learning interest, learning initiative and positivity, and practical ability
Curran et al., 2015, Canada [14]	RCT	Third year medical students/*n* = 66	High-fidelity simulator group (*n* = 31) vs. Low-fidelity simulator group (*n* = 35)	Integrated skills performance; Teamwork behaviors; Participant satisfaction scores; Confidence survey	No difference in skill performance (*p* = 0.45) and teamwork behavior (*p* = 0.144); Improved satisfaction scores in high-fidelity group, *p* < 0.01; Improved confidence in high-fidelity group, *p* < 0.01
Nimbalkar et al., 2015, India [10]	RCT	Undergraduate students/*n* = 101	High-fidelity simulator group (*n* = 50) vs. Low-fidelity simulator group (*n* = 51)	Neonatal resuscitation knowledge by written test; Skills performance by Megacode; Long-term outcomes (3 months)	Improved knowledge scores in high-fidelity group, *p* < 0.05; No difference in skill performance, *p* = 0.13
Chen et al., 2015, China [24]	RCT	Medical students/*n* = 40	High-fidelity simulator group (*n* = 20) vs. traditional training group (*n* = 20)	Knowledge test; Skills performance test; Satisfaction survey	Improved knowledge scores in high-fidelity group, *p* < 0.05; Improved knowledge scores in high-fidelity group, *p* < 0.01; Improved satisfactory in learning interest, learning initiative and positivity, practical ability, Teamwork awareness, critical thinking, and clinical thinking
Rubio-Gurung et al., 2014, France [25]	RCT	Level 1 and Level 2 maternities/*n* = 12	High-fidelity simulator group (*n* = 6) vs. No intervention group (*n* = 6)	Technical scores (TS); Team performance scores (TPS)	Improved in median TS and TPS in the Intervention group than in the Control group after the training sessions, *p* < 0.05
Cheng et al., 2013, Canada [13]	RCT	Interprofessional health careteams/*n* = 90	Non-scripted debriefing, low-fidelity simulator (*n* = 23) vs. scripted debriefing, low-fidelity simulator (*n* = 22) vs. non-scripted debriefing, high-fidelity simulator (*n* = 23) vs. scripted debriefing, high-fidelity simulator (*n* = 22)	Medical knowledge by multiple choice question (MCQ) test; Team clinical management by Clinical Performance Tool (CPT); Team leader’s behavioral performance by Behavioral Assessment Tool (BAT)	No difference in MCQ (*p* = 0.67), BAT (*p* = 0.72), and CPT (*p* = 0.1) between high-fidelity group and low-fidelity group after debriefing
Campbell et al., 2009, Canada [26]	RCT	First-year family medicine residents/*n* = 15	High-fidelity simulator group (*n* = 8) vs. Low-fidelity simulator group (*n* = 7)	Experience rating for Knowledge test; Megacode for performance	Improved knowledge scores in high-fidelity group, *p* < 0.05; Improved skill performance in high-fidelity group, *p* < 0.05
Lee et al., 2012, USA [27]	RCT	2nd-4th year emergency medicine residents/*n* = 27	High-fidelity simulator group (*n* = 12) vs. traditional training group (*n* = 15)	Knowledge, skill performance; Confidence survey	Improved knowledge, skill and confidence scores from baseline to final assessment in high-fidelity group, *p* < 0.05
Finan et al., 2012, Canada [28]	RCT	Neonatal trainees/*n* = 16	High-fidelity simulator group (*n* = 8) vs. Low-fidelity simulator group (*n* = 8)	NRP performance scores; Non-technical team performance	No difference between high-fidelity group and low-fidelity group in NRP performance scores (*p* = 0.17) or non-technical skills performance between groups (*p* = 0.52)
Thomas et al., 2010, USA [12]	RCT	Residents/*n* = 34	High-fidelity simulator + team training group (*n* = 10) vs. Low-fidelity simulator + team training group (*n* = 9) vs. Low-fidelity simulator group (*n* = 15)	Teamwork outcomes; Performance score and resuscitation duration	Improved teamwork event behaviors in high-fidelity groups (*p* = 0.004); No difference between high-fidelity team training and low-fidelity team training group in NRP performance (*p* = 0.999) or resuscitation duration (*p* = 0.452)

### Description of the intervention in the studies

Of the 15 included studies, 12 studies reported use of the manikin with high-fidelity simulation. SimBaby (six studies) and SimNewB (five studies) were most widely applied. All studies reported the training content, which ranged from didactic lectures and simulated resuscitation training based on the NRP guideline to scenario-based practices. Seven studies described the introducers, and only two of them [12, 29] were experienced NRP introducers. Most studies (14) implemented the scenario into the simulated resuscitation. Nine studies reported specific scenarios, including full-term and preterm neonate scenarios with vital signs responsive to hypoxic events and interventions. About half of the studies (eight) described a debriefing session, but only four studies [11, 13, 22, 25] designed the debriefing involving both the residents and instructors/trainers, and was conducted immediately after the scenario training. One study [21] designed concurrent debriefing to facilitate learning throughout the practice session, one study [20] designed the debriefing to be performed by residents, and two studies [12, 27] designed the debriefing to be performed by instructors/trainers with the video records. Eleven studies reported the learning time or duration of the intervention. Details of the intervention of the included studies are shown in Additional file 2: Table S1.

### Quality of the studies

**Fig. 2:**
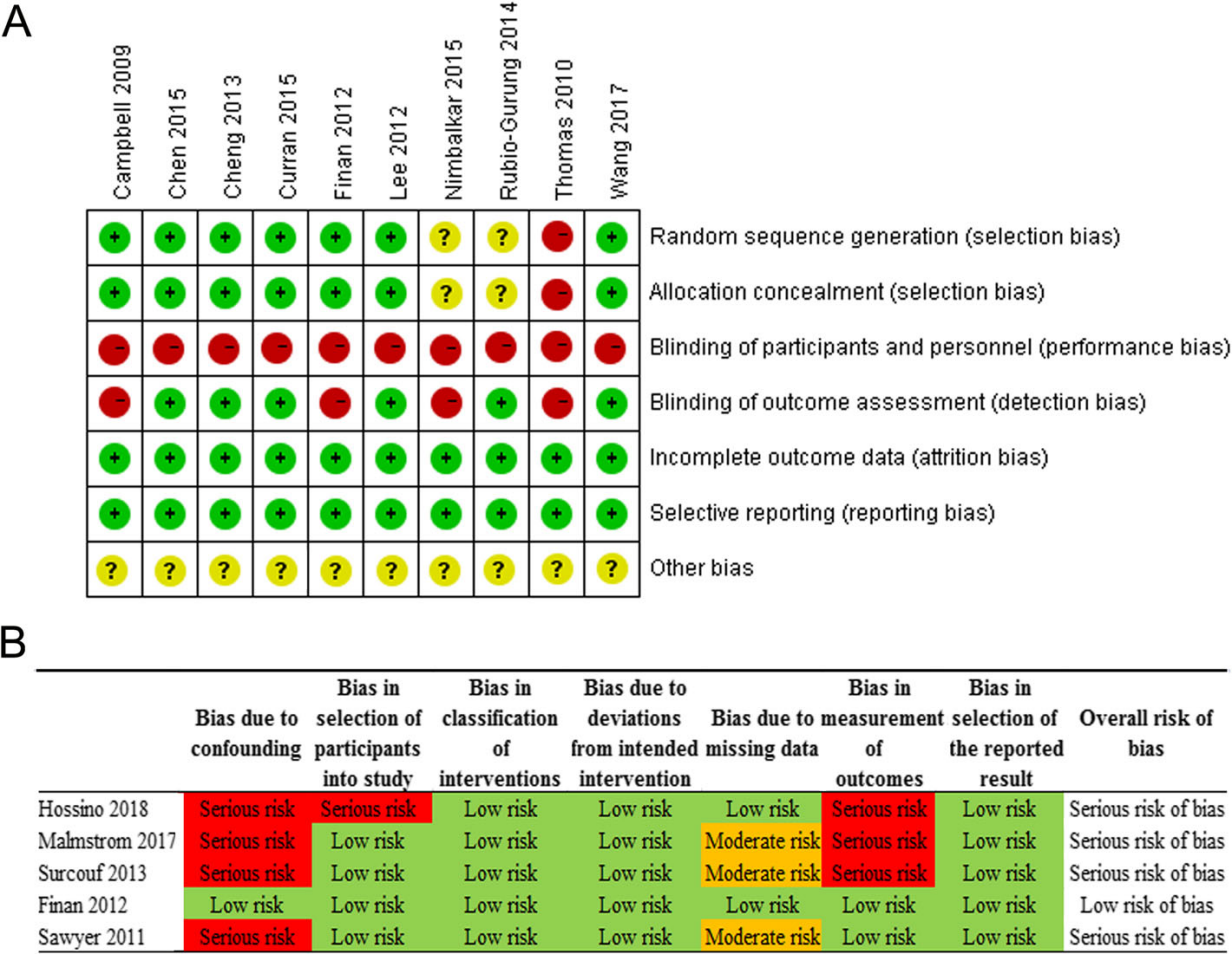
Quality evaluation and bias assessment of the included studies. **a** Quality evaluation and bias assessment of the randomized controlled trials; **b** Quality evaluation and bias assessment of the non-randomized controlled trials

Ten RCTs and five single arm pre-post studies were included. The risk of bias assessment of RCTs is summarized in Fig. 2a. Performance bias existed in all RCTs because participant blinding to the simulator is impossible. Additionally, four study [14, 23, 24, 27] analyzed data of the subjective outcome measurement such as confidence and satisfaction survey, which resulted in the bias of blinding of the outcome assessment. There was incomplete evidence of random sequence generation and allocation concealment in three studies [13, 14, 24]. Figure 2b shows the assessment of the risk of bias of single arm pre-post studies. Only one study [21] was of high quality with a low risk of bias, whereas four [11, 19, 20, 22] were of low quality with a serious risk of bias. Of these low quality studies, none specified whether the included population had prior neonatal clinical experience (bias due to confounders). One of them included general residents (unspecified medical subspecialty) rather than residents in pediatrics or a resuscitation-related medical subspecialty (bias in the selection of participants). Three of them missed a small amount of data in the outcome assessment (bias due to missing data). Finally, three of them used a subjective outcome measurement (bias in the measurement of outcomes).

### Efficacy of high-fidelity simulation

**Fig. 3:**
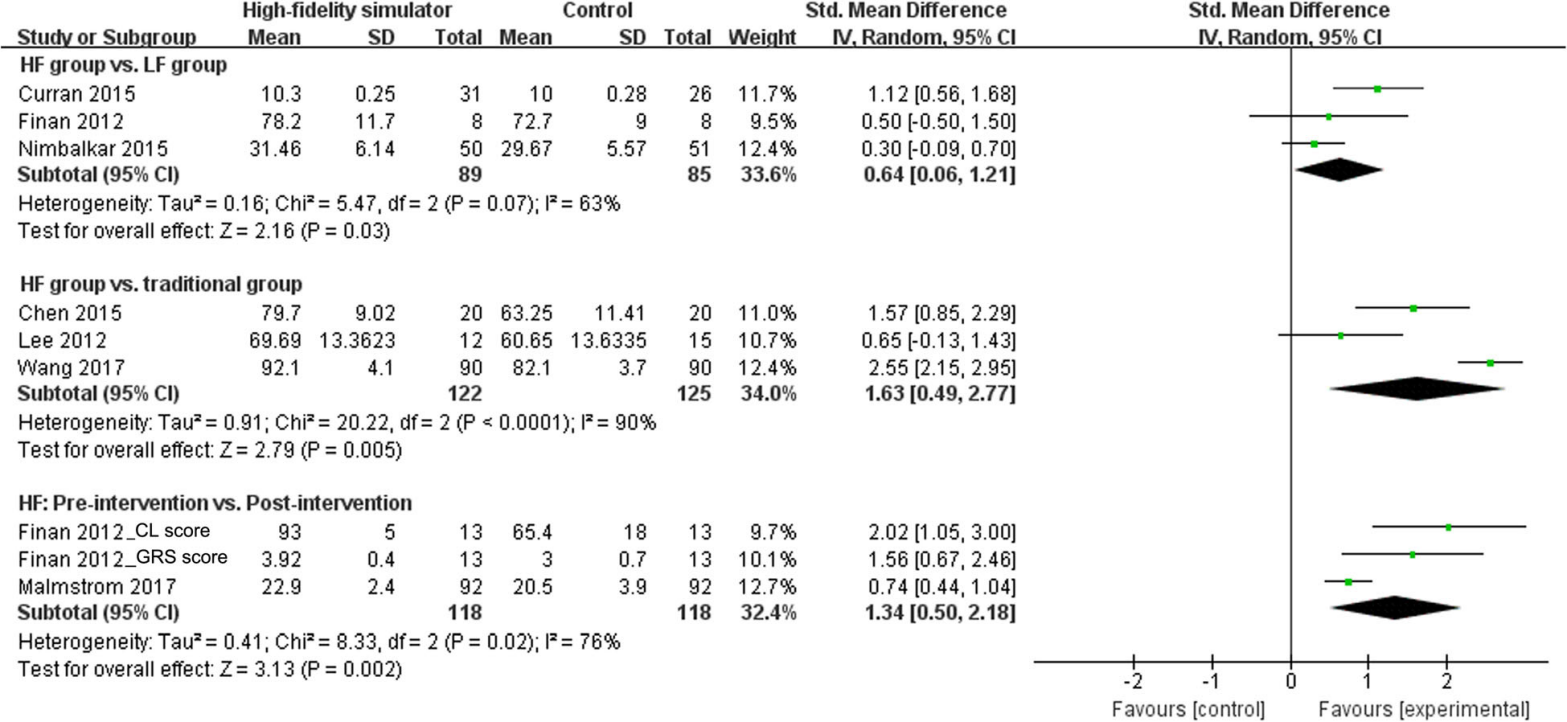
Forest plot showing the efficacy of high-fidelity simulation training in skill performance. HF: high-fidelity, LF: high-fidelity, CL score: intubation checklist, GRS score: global rating scale score

Five single-arm pre-post studies assessed the effect of high-fidelity simulation on neonatal resuscitation outcomes. Four of them measured the individual scores, whereas one [22] measured the team scores (pairs of two) pre-post intervention. In all pre-post studies, high-fidelity simulation had a positive effect on short-term outcome measures, including knowledge, skill performance, teamwork performance, and confidence. The pooled results by meta-analysis showed a large benefit with high-fidelity simulation in skill performance (SMD 1.34; 95% CI 0.50–2.18) and the evidence of heterogeneity with an I^2^ = 76% was also shown (Fig. 3). However, one high-quality study [21] performed a real-life test after the intervention and in addition to the simulation test, and they reported that the improved performance in the simulation environment after the intervention may not be transferable to the clinical setting.

**Fig. 4:**
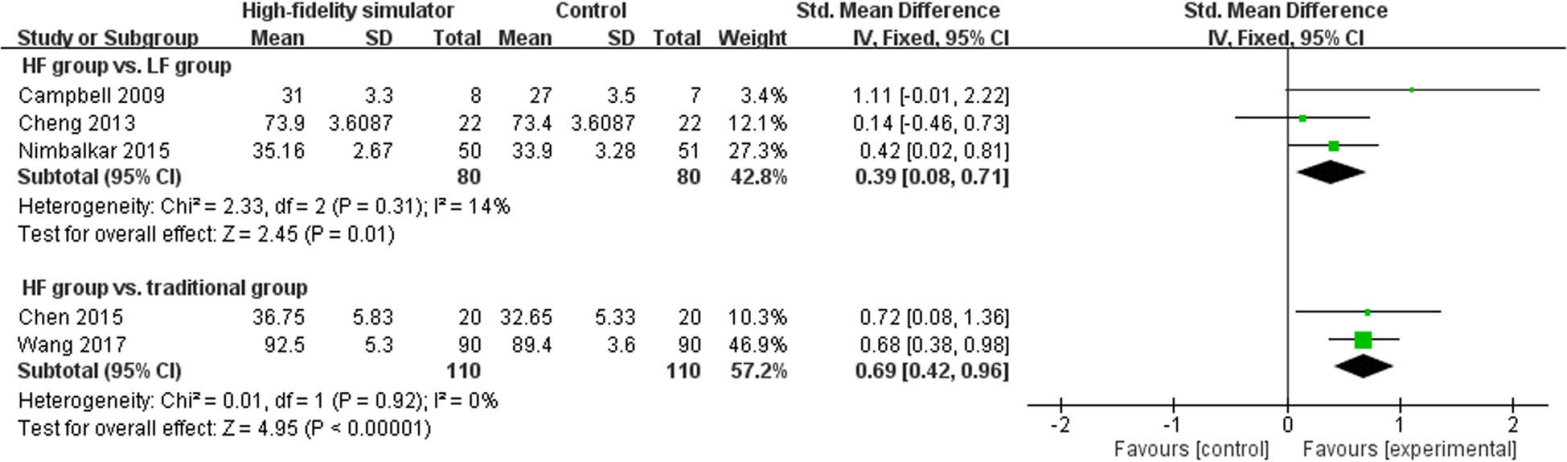
Forest plot showing the efficacy of high-fidelity simulation training in knowledge. HF: high-fidelity, LF: high-fidelity

Three RCTs [23, 24, 27] investigated the efficacy of high-fidelity simulation compared with traditional training. The pooled results by meta-analysis showed a large benefit with high-fidelity simulation when compared with traditional training in skill performance (SMD 1.63; 95% CI 0.49–2.77) (Fig. 3) and a moderate benefit in neonatal resuscitation knowledge (SMD 0.69; 95% CI 0.42–0.96) (Fig. 4). There was evidence of heterogeneity with an I^2^ = 90% in the comparison of skill performance (Fig. 3). Moreover, a RCT grouped by maternities performed by Rubio-Gurung et al. [25] found improved skill performance in the intervention group compared with the control group after the training sessions.

### High-fidelity versus low-fidelity simulation

Seven RCTs investigated the efficacy of high-fidelity versus low-fidelity simulation in training. Three RCTs [10, 14, 28] measured the skill performance and found a moderate benefit with high-fidelity simulation when compared with low-fidelity simulation (SMD 0.64; 95% CI 0.06–1.21) (Fig. 3). There was evidence of heterogeneity with an I^2^ = 63% in the comparison of skill performance (Fig. 3). However, an RCT of team training conducted by Thomas et al. [12] did not find a significant difference between high-fidelity and low-fidelity training teams in the neonatal resuscitation skill performance. Three RCTs [10, 13, 26] measured the neonatal resuscitation knowledge after simulation, and the pooled results by meta-analysis showed a small benefit with high-fidelity simulation when compared with low-fidelity simulation (SMD 0.39; 95% CI 0.08–0.71) (Fig. 4). Additionally, teamwork performance was an important outcome measure in four RCTs. Thomas et al. [12] found improved teamwork event behaviors in high-fidelity groups when compared with low-fidelity groups. Contrarily, the other three studies [13, 14, 28] did not find a significant difference between the two groups.

In terms of the long-term outcomes, one RCT [10] found no differences between the two groups, neither in the level of neonatal resuscitation knowledge nor in skill performance after 3 months of training course, although there was a significant difference in short-term outcomes. Conversely, Rubio-Gurung et al. [25] did not find a difference between the two groups immediately after the intervention, but there was a significant improvement in the high-fidelity group after a median of 107 days in both technical and team performances.

## Discussion

### Strengths and limitations

To our knowledge, this is the first systematic review to assess the qualities of current trials concerning the role of high-fidelity simulation in neonatal resuscitation training and the first meta-analysis of its kind to pool the current results of its benefit. The strengths of this study include the following: (1) it was a comprehensive literature search of clinical trials; (2) it comprised a quality evaluation of current studies concerning the role of high-fidelity simulation in neonatal resuscitation training, especially using the ROBINS-I tool, to assess the methodological quality of non-RCTs in this field; (3) it included separate comparisons of meta-analyses according to outcome and study design; and (4) it compared the instructors, scenarios implemented, and debriefing performed among the included studies.

Several limitations in this review should be addressed. First, significant heterogeneity in the meta-analysis of skill performance was found, which might result from the different educational setting, included population groups, and outcomes measures among studies. Furthermore, the number of included studies limited our ability to investigate these factors. Second, although we extensively searched databases, there were a limited number of studies. Each meta-analysis included only two or three studies. Two RCTs [13, 25] were excluded from meta-analysis because their outcome measures were based on the maternities or teams rather than the individuals. Third, 10 of the included studies were performed in North America, and five were conducted in other parts of the world. This imbalance of countries and different target populations might result in selection bias of population. Fourth, the confidence and satisfaction assessments among the included studies were based on various standards or questionnaires, which were objective and might lack reliability. Finally, although the NRP standard was described as the training standard in the included studies, some confounding factors exist among them, such as the competency of instructors [30], complexity and length of the scenarios, and content of a debriefing session. Therefore, the quality assurance process of simulation-based training might vary between different NRP instructors and settings.

### Comparison with previous reviews and efficacy of short-term outcomes

High-fidelity simulation has been applied recently in the education of emergency medicine [31], pediatrics [8], and resuscitation [32]. A previous systematic review in 2014 [33] summarized the results of simulation-based neonatal resuscitation teaching based on RCTs, but only two RCTs concerning the use of high-fidelity simulation in neonatal resuscitation training were included in that review. Similarly, another systematic review in that year [34] identified two trials of high-fidelity simulation-based neonatal resuscitation teaching. However, no recommendation was made about which level of fidelity simulation is more effective in neonatal resuscitation according to previous reviews. In this study, we found that high-fidelity simulation training was more effective both in the improvements of knowledge scores and skill performance. Furthermore, the degree of benefits was lower in short-term outcomes when comparing high-fidelity to low-fidelity simulation than when comparing high-fidelity to no simulation. Only small to moderate benefits were found in comparing high-fidelity to low-fidelity simulation, which was similar to the results of a previous meta-analysis on high-fidelity simulation-based training of PALS performed by Cheng et al. [9]. These low-degree benefits might result from the limitation of simulation. As simulation is just a tool, experienced facilitators/instructors, the case scenarios, and debriefing sessions are all important components in the efficacy of training. Both the low-fidelity and high-fidelity simulation trainings should be controlled by the instructors with case scenarios [5, 35]. Consequently, it is relatively limited to improve the benefit through only increasing the fidelity of simulation. The larger benefits may be achieved in training efficacy when guaranteeing the high-quality matching of simulation with experienced instructors, the scenarios, and debriefing.

Teamwork and communication training is a key way to improve resuscitation performance [36]. Simulation training is used extensively in the training of effective teamwork and communication skills as a safe and high-quality method [3]. Hence, the use of high-fidelity simulation-based training is considered effective in teamwork performance because the training focuses on communication improvement, situational awareness, and task distribution [12, 37]. A previous review in 2011 provided an overview of high-fidelity simulation-based training in the NRP and PALS, which concluded that high-fidelity simulation engenders improvements in team communication [5]. In our study, four RCTs analyzed the teamwork efficacy of high-fidelity simulation-based training, but a positive result was shown in only one study. Because of the different standard and unclear content of teamwork performance measurements in the included studies, a meta-analysis cannot be performed. Therefore, more studies are needed to identify whether high-fidelity simulation-based neonatal resuscitation training is more effective compared with that of no simulation or low-fidelity simulation in teamwork performance improvement.

### Skill retention and translated efficacy

The training time and duration are key points for the long-time retention of improved skills [38]. Only two trials in our review measured the long-time retention of benefits, and their results were inconsistent. In the Nimbalkar et al.’s study, the total learning time was 18 h over a 3-day period, and a negative result was found after 3 months of training. On the contrary, Rubio-Gurung et al. found a positive long-time retention after 107 days of training with continuous learning for 1 month (four hours daily). Therefore, more trials are needed to find out factors related to long term outcomes and professionals in this field need to stress on researchers to investigate long term and clinical outcomes.

There has been no assessment of the translated clinical performances in real-life situations after high-fidelity simulation-based training in the included studies, except for one. This one study [28] did not find a positive result in the clinical translation of simulation-based benefits. We speculated that this lack of evaluation of translated efficacy might be because of the high risk and difficulty of skill measurement in neonates of the clinic. However, SBE has been recommended by neonatal resuscitation guidelines, and the evaluation of translated efficacy in real-life situations must become an essential part of high-fidelity simulation-based training. Interestingly, regarding evaluations of the effects of SBE on gastrointestinal endoscopy, we found that many studies used patient-related outcomes such as the cecal intubation rate in colonoscopy or major complications as the evaluation of translated efficacy [39]. Therefore, in future studies, some patient-related indicators such as the success rate of endotracheal intubation can be considered as the evaluations of translated efficacy in real-life clinical practice.

## Implications and conclusions

Our findings have important implications for current practice and future studies. High-fidelity simulation-based neonatal resuscitation training is effective on short-term outcomes, but the benefits are only small to moderate when compared with low-fidelity simulation training. The learning cycles of adults consist of initial experience, opportunity to reflect (such as debriefing), conceptualization of new knowledge, and experimentation with new skills [40]. This principle indicated that debriefing is a critical phase to determine the efficacy of high-fidelity simulation training [41]. Nevertheless, only four included studies reported a structured reflection/debriefing in both students and trainers. Hence, an immediate and meaningful debriefing session should be introduced in each high-fidelity simulation training in the future, and it should include three phases of descriptive, analysis, and application both in students and trainers [5]. In addition, the complexity and length of the scenarios used in the teaching session, and the competency and experience of instructors are critical influential factors of the efficacy of high-fidelity simulation training. However, these factors are not controlled well in current studies. Thus, in future studies, a validated assessment tool (including assessment of instructors, scenarios, and debriefings) needs to be developed to standardize the design and implementation of high-fidelity simulation-based NRP training, and achieve larger benefits of training.

In the meta-analysis, improvements of efficacy were shown in both resuscitation knowledge and skill performance immediately after training, although the evidence was limited by the small number of trials and different NRP training settings among the studies. However, in current studies, the long-term retention of benefits is controversial, and these benefits may not transfer to real-life situations. Given that SBE has been recommended by neonatal resuscitation guidelines, more high-quality RCTs should be performed to validate its efficacy and to explore the outcomes of long-term retention, translated efficacy to the real-life environment, and teamwork performance.

## Additional files

Additional file 1: Search terms. (DOCX 22 kb)

Additional file 2: **Table S1.** Details of the intervention of the included studies. (XLSX 14 kb)

## Data Availability

All raw data used in this systematic review were extracted from available published articles.

## Abbreviations

CBM: Chinese biomedical literature database
CI: Confidence interval
CNKI: Chinese national knowledge infrastructure
HF: High-fidelity
LF: Low-fidelity
NRP: Neonatal resuscitation program
PALS: Pediatric advanced life support
SBE: Simulation-based education
SMD: Standardized mean difference
