# Supplementary material for: Educational efficacy of high-fidelity simulation in neonatal resuscitation training: a systematic review and meta-analysis
Source: BMC Med Educ. 2019 Aug 29;19:323. doi: 10.1186/s12909-019-1763-z (PMC6716944; doi:10.1186/s12909-019-1763-z)
Supplement: Supplementary file 1 — Search terms. (DOCX 22 kb) [file 12909_2019_1763_MOESM1_ESM.docx]

**Search Terms:**

(((((simulation) OR manikin) OR mannequin)) AND ((Neonatal resuscitation) OR Infant resuscitation)) AND (((Training) OR Teaching) OR Education)

**PubMed**

#1 MeSH Terms search: (((((resuscitation[MeSH Terms]) AND ((infant[MeSH Terms]) OR neonatal[MeSH Terms])) AND (((Simulation Training[MeSH Terms]) OR Education[MeSH Terms]) OR Training[MeSH Terms])) AND (((patient simulation[MeSH Terms]) OR High Fidelity Simulation Training[MeSH Terms]) OR Simulation Training[MeSH Terms])))

#2 All fields search: (((((simulation) OR manikin) OR mannequin)) AND ((Neonatal resuscitation) OR Infant resuscitation)) AND (((Training) OR Teaching) OR education)

#3 Search #1 OR #2

**The search of other databases was based on all fields search.**
